# Short-term mortality in older medical emergency patients can be predicted using clinical intuition: A prospective study

**DOI:** 10.1371/journal.pone.0208741

**Published:** 2019-01-02

**Authors:** Noortje Zelis, Arisja N. Mauritz, Lonne I. J. Kuijpers, Jacqueline Buijs, Peter W. de Leeuw, Patricia M. Stassen

**Affiliations:** 1 Department of Internal Medicine and Gastroenterology, Zuyderland Medical Centre, Heerlen, The Netherlands; 2 CARIM School for Cardiovascular Diseases, Maastricht University Medical Centre, Maastricht University, Maastricht, The Netherlands; 3 Department of Internal Medicine, Division of General Internal Medicine, Section Acute Medicine, Maastricht University Medical Centre, Maastricht University, Maastricht, The Netherlands; 4 School of CAPHRI, Maastricht University Medical Centre, Maastricht University, Maastricht, The Netherlands; Nord University, NORWAY

## Abstract

**Background:**

Older emergency department (ED) patients are at risk for adverse outcomes, however, it is hard to predict these. We aimed to assess the discriminatory value of clinical intuition, operationalized as disease perception, self-rated health and first clinical impression, including the 30-day surprise question (SQ: “Would I be surprised if this patient died in the next 30 days” of patients, nurses and physicians. Endpoints used to evaluate the discriminatory value of clinical intuition were short-term (30-day) mortality and other adverse outcomes (intensive/medium care admission, prolonged length of hospital stay, loss of independent living or 30-day readmission).

**Methods:**

In this prospective, multicentre cohort study, older medical patients (≥65 years), nurses and physicians filled in scores regarding severity of illness and their concerns (i.e. disease perception and clinical impression scores) immediately after arrival of the patient in the ED. In addition, patients filled in a self-rated health score and nurses and physicians answered the SQ. Area under the curves (AUCs) of receiver operating characteristics (ROCs) were calculated.

**Results:**

The median age of the 602 included patients was 79 years and 86.7% were community dwelling. Within 30 days, 66 (11.0%) patients died and 263 (43.7%) patients met the composite endpoint. The severity of concern score of both nurses and physicians yielded the highest AUCs for 30-day mortality (for both 0.75; 95%CI 0.68–0.81). AUCs for the severity of illness score and SQ of nurses and physicians ranged from 0.71 to 0.74 while those for the disease perception and self-rated health of patients ranged from 0.64 to 0.69. The discriminatory value of the scores for the composite endpoint was lower (AUCs ranging from 0.60 to 0.67). We used scores that have not been previously validated which could influence their generalisability.

**Conclusion:**

Clinical intuition,—disease perception, self-rated health and first clinical impression—documented at an early stage after arrival in the ED, is a useful clinical tool to predict mortality and other adverse outcomes in older ED patients. Highest discriminatory values were found for the nurses’ and physicians’ severity of concern score. Intuition may be helpful for the implementation of personalised medical care in the future.

## Introduction

After an emergency department (ED) visit, older patients experience high rates of hospitalisation, functional decline, readmission and mortality [1, 2]. Important decisions concerning diagnostics and treatment have to be made within a short timeframe during an ED visit. Therefore, accurate identification of high risk patients is essential for optimal clinical care and safety. At this moment, reliable ways to predict adverse outcomes in older patients with generic problems are lacking [3, 4].

Clinical intuition can be a way to predict adverse outcomes in older ED patients. Indeed, the clinical impression of both nurses and physicians was associated with adverse outcomes [5–9]. The disease perception by patients [10] and self-rated health [11–13] were associated with both morbidity and mortality as well. In addition, the surprise question (SQ) “Would I be surprised if this patient died in the next 12 months?” was found to predict adverse outcomes, although its reported accuracy varied widely [14, 15]. Most studies regarding clinical intuition were performed in younger patients [6, 8–10], clinical settings other than the ED (e.g. admission units [6, 16] and Intensive Care Units (ICUs) [8]), in selected groups of patients (e.g. patients with sepsis, cancer, renal failure or non-specific complaints [7, 9, 14]), and they used long-term mortality as endpoint [11, 12, 14]. Moreover, most studies were performed with professionals who had access to results of physical examination and diagnostic test results and therefore, the *first* clinical impression was not tested.

Therefore, the value of clinical intuition; disease perception, self-rated health and *first* clinical impression, in predicting short-term mortality and other adverse outcomes remains unknown. We hypothesize that clinical intuition predicts adverse outcomes in older ED patients. To test this hypothesis, we set up a prospective multicentre cohort study to assess the discriminatory value of clinical intuition with respect to 1) short-term (30-day) mortality and 2) other adverse outcomes such as ICU or medium care unit (MCU) admission, prolonged hospital stay (LOS), loss of independent living and unplanned readmission.

## Materials and methods

### Study design and setting

This study is part of the Risk Stratification in the Emergency Department in Acutely ill Older Patients (RISE UP) study, a prospective multicentre observational cohort study that aims to identify predictors of adverse outcomes in older medical ED patients. This study was conducted at the EDs in Zuyderland Medical Centre (MC) and Maastricht University Medical Centre+ (MUMC+) in The Netherlands. These hospitals are both teaching hospitals, the first providing secondary, the second providing both secondary and tertiary care. This study was approved by the medical ethics committees of Zuyderland MC and MUMC+ (NL55867.096.15) and is registered on clinicaltrials.gov (NCT02946398).

### Study population

All older (≥65 years) medical ED patients who were assessed and treated under supervision of internists or gastroenterologists were eligible for inclusion. Internal medicine residents and emergency physicians included the patients in Zuyderland MC from July 2016 until February 2017 and in MUMC+, from September 2016 until February 2017. All patients or their legal representatives signed an informed consent form before study entry. We excluded patients who already participated in the study, who were unable to speak Dutch, German or English or who were admitted to a ward of another specialty than internal medicine or gastroenterology. We assumed that in certain circumstances the physicians would give priority to emergency care and that not all possible candidates could be asked to participate in this study. This turned out to be true, and therefore, the patients formed a convenience sample. Because of this finding, we evaluated possible selection bias by analysing data of non-included patients.

### Data collection and measurements

Data were collected from the patients’ electronic medical records and questionnaires. Immediately after arrival, all participants received a questionnaire, which was filled in by the patient or caregiver. The attending nurse and physician completed a questionnaire at the same moment, immediately after obtaining informed consent and before history taking, physical examination and without knowledge of any diagnostic results. All respondents filled in the questionnaires independent of each other to ensure blinding of the results. Questions about disease perception, self-rated health and first clinical impression including the SQ were scored using a Likert scale from 1 to 5 (S1 File). In this manuscript, we will refer to these questions as scores, except for the SQ.

The questionnaires of patients, nurses and physicians consisted of three identical questions: 1) “How severely ill are you/do you find this patient?”(severity of illness score) and 2) “Are you concerned about your/her/his condition?” (severity of concern score) 3) “Are you concerned about loss of independency after this hospital visit?” for patients and “Do you think this patients will lose independency?” for nurses and physicians (loss of independency score). Patients or their family members/caregivers had to answer one additional question: “How would you describe your health before your visit to the ED?” (self-rated health score [13]). Nurses and physicians answered three additional questions. The first additional question was the 30-day SQ [17]: “Would you be surprised if this patient died in the next 30 days?”. The second question was: “Do you think this patient will be admitted for more than 7 days?” (length of hospital stay (LOS) score). Third, the number of years of experience was asked.

We collected data on demographics, living situation, comorbidities (according to the Charlson Comorbidity Index [18]) and cognitive function from medical records. Functional status was assessed using a questionnaire to calculate the Katz Activities of Daily Living (ADL) index score [19] in all hospitalised patients. We recorded the main reason for the ED-visit according to the International Classification of Diseases (ICD)-10 system [20].

To analyse possible selection bias, for 200 patients who were not included in this study, we collected the abovementioned data, except for data on the questionnaires and the Katz-ADL index score. Demographics were collected for all possible candidates during the study period.

### Primary and secondary outcome measures

The primary endpoint to evaluate the discriminatory value of clinical intuition was 30-day all-cause mortality, for the severity of illness, severity of concern, SQ and self-rated health score. The secondary endpoint was a composite endpoint consisting of admission to ICU/MCU, prolonged LOS (>7 days), loss of independent living and unplanned readmission within 30 days after discharge. This composite endpoint was used to assess the value of the severity of illness, severity of concern and self-rated health score. Prolonged LOS and loss of independent living were calculated as single secondary endpoints as well. For the analysis of prolonged LOS, patients who were not admitted to the hospital or who died within seven days during hospital stay were excluded. Loss of independent living was defined as discharge to a nursing home/hospice/revalidation clinic or palliative care in previously community dwelling patients. For the analysis of loss of independent living, only patients who were discharged alive and who were not fully dependent (i.e. living in nursing home) before admission were included. Patients who died during the first admission or within 30 days after discharge before being readmitted were excluded in the analysis of readmissions.

### Statistical analysis

We performed a descriptive analysis of baseline characteristics of included patients and endpoints. Continuous variables were reported as means with standard deviations or medians with interquartile ranges (IQRs) and categorical variables as proportions. In case of missing values, valid percentages were used.

The discriminatory value of the disease perception, self-rated health and clinical impression scores was analysed by calculating the area under the curves (AUCs) of receiver operating characteristics (ROCs) with 95% confidence intervals (CIs). An AUC of 0.9–1.0 was considered as being excellent, 0.8–0.9 very good, 0.7–0.8 good, 0.6–0.7 sufficient and 0.5–0.6 as bad accuracy [21]. The method of DeLong [22] was used to test for significant differences between AUCs of correlated patients’, nurses’ and physicians’ scores.

Sensitivity, specificity, positive predictive values (PPVs) and negative predictive values (NPVs) were calculated for different cut-off values of the scores. An optimum cut-off value was chosen based on the value being closest to the upper left corner of the AUC. When two values were equally distanced, the value with the highest Youden’s Index [21] was selected. Both positive and negative likelihood ratios (LRs) were calculated. For the SQ, loss of independency score and LOS score of nurses and physicians, score 1 and 2 were considered negative and score 4 and 5 positive. Score 3, “I don’t know”, was not included in the analysis.

The interrater reliability of the scores was calculated using the intraclass correlation coefficient (ICC) using a two-way random model for patients, nurses and physicians [23]. An ICC under 0.40 was considered poor, between 0.40–0.59 fair, 0.60–0.74 good and between 0.75–1.00 excellent [24]. LRs for 30-day mortality were calculated for cases in which patients, nurses and physicians agreed or disagreed.

All analyses were performed using IBM SPSS Statistics for Windows, Version 24.0 (IBM Corp., Armonk, N.Y., USA) and R version 3.4.4. P-values <0.05 were considered statistically significant.

## Results

### Study population

**Fig 1:**
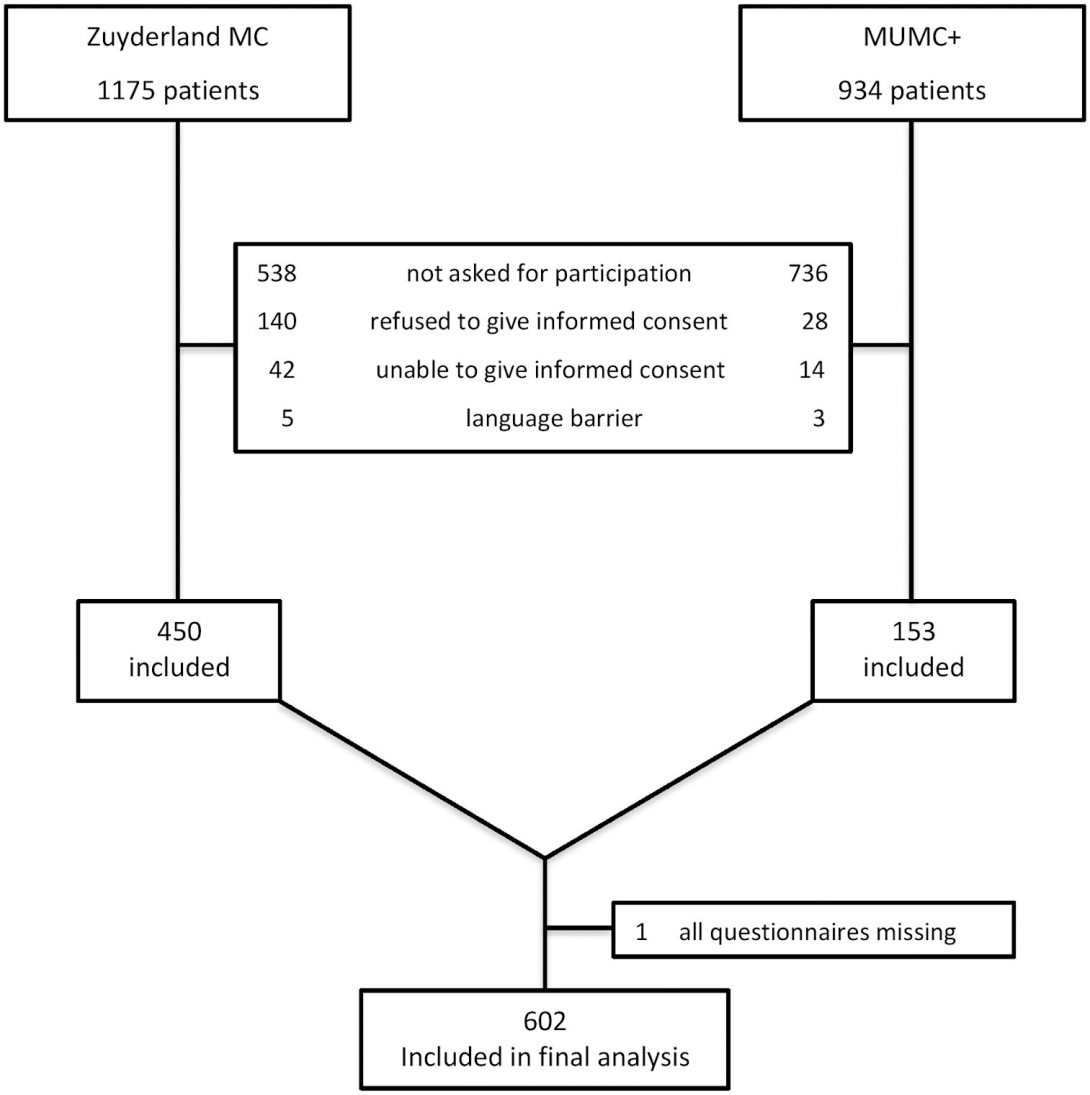
Flowchart of Zuyderland MC and MUMC+ patients.

During the study period, 2109 older patients (1175 patients in Zuyderland MC and 934 patients in MUMC+) visited the ED and were treated by internists or gastroenterologists (Fig 1). In total, 1506 (71.4%) patients were not included because 1) they were not asked to participate (n = 1274, 60.4%) 2) they refused to give informed consent (n = 168, 8.0%), 3) they were unable to give informed consent (n = 56, 2.7%) or 4) there was a language barrier (n = 8, 0.4%). In total, 603 (28.6%) patients were enrolled in this study. Since for one patient all questionnaires were missing, 602 patients were included in the final analysis.

### Patient characteristics and questionnaires

The median age of the study population was 79 years (73–85) and 51.7% were male (Table 1). Most patients were community-dwelling (86.7%) and the median Katz-ADL score was 0 [0–2]. Nurses and physicians had a median professional experience of 3 (IQR: 1–15) and 2 (IQR 1–3) years, respectively.

**Table 1:** Characteristics of patients and professionals^a^.

	All patients N = 602
Demographics	
Age (years), median (IQR)	79 (73–85)
Male	311 (51.7)
Living situation	
Community-dwelling	522 (86.7)
Nursing- and care home	50 (8.3)
Other	30 (5.0)
Comorbidity and functional status	
Charlson comorbidity index score, median (IQR)	2 (1–3)
Katz-ADL index score^b^, median (IQR)	0 (0–2)
Reason for ED visit (ICD-10)	
Infectious diseases	176 (29.2)
Diseases of the digestive system	156 (25.9)
Diseases of the circulatory system	55 (9.1)
Neoplasms	52 (8.6)
Endocrine, nutritional and metabolic diseases	31 (5.1)
Diseases of the respiratory system	30 (5.0)
Diseases of the blood and blood-forming organs	28 (4.7)
Diseases of the genitourinary system	28 (4.7)
Miscellaneous	46 (7.6)
Admission	478 (79.4)

Abbreviations: ADL, Activities of Daily Living; ICD-10, International Classification of Diseases-10; IQR, interquartile range

^a^ Values are numbers (percentages) unless stated otherwise. Incomplete data for: Katz-ADL index score (n = 1), years of experience nurse (n = 178), years of experience physician (n = 34).

^b^Katz-ADL index score was calculated for all hospitalized patients

Patients or their caregivers filled in 594 (99.0%) questionnaires (91.1% completely), nurses 568 (94.7%; 99.3% completely) and physicians 597 questionnaires (99.5%; 99.8% completely).

### Predictive ability of the scores for 30-day mortality and the composite endpoint

**Fig 2:**
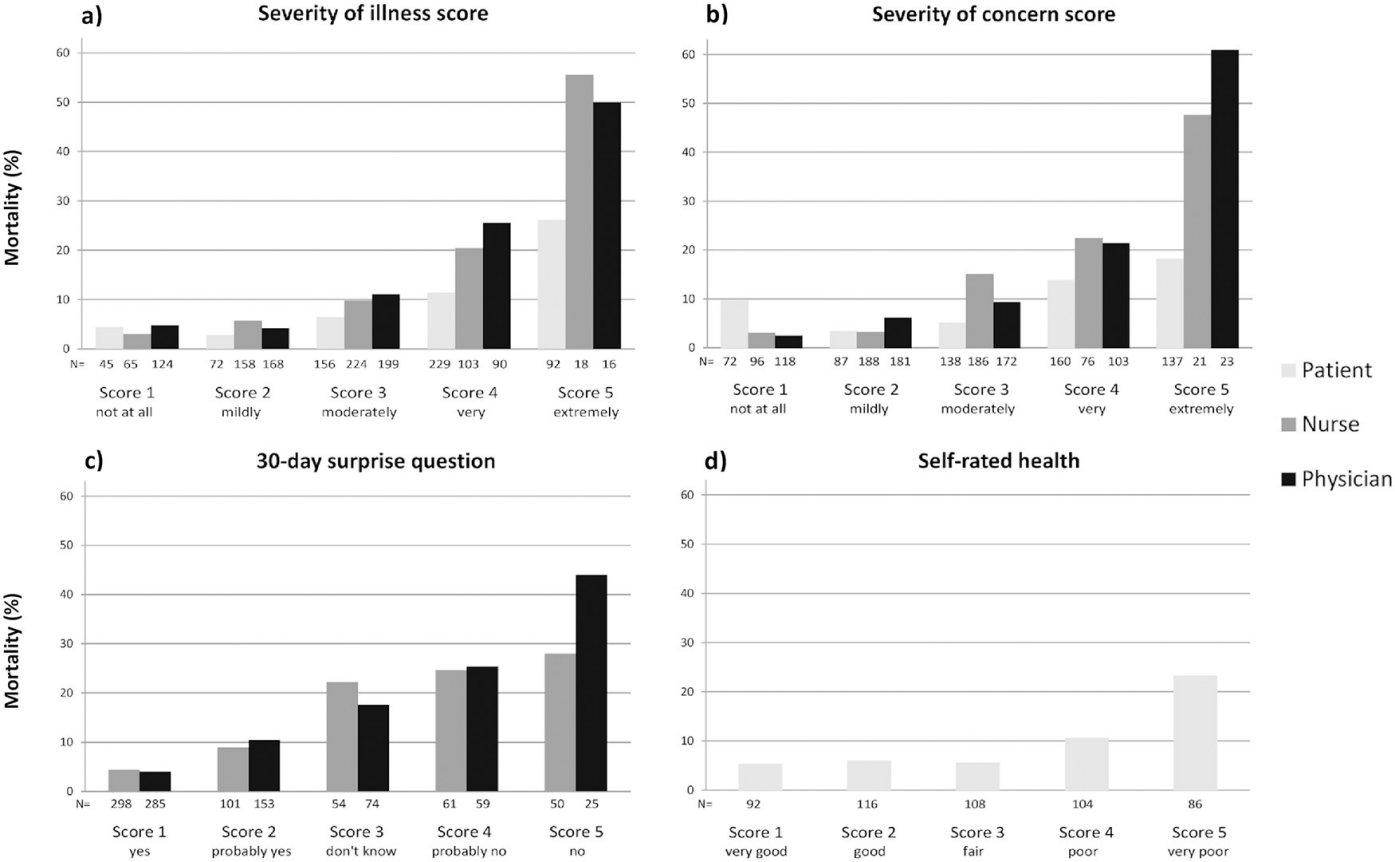
Association between the scores and 30-day mortality for the severity of illness score (a), severity of concern score (b), 30-day surprise question (c) and self-rated health score (d) for patients, nurses and physicians.

**Fig 3:**
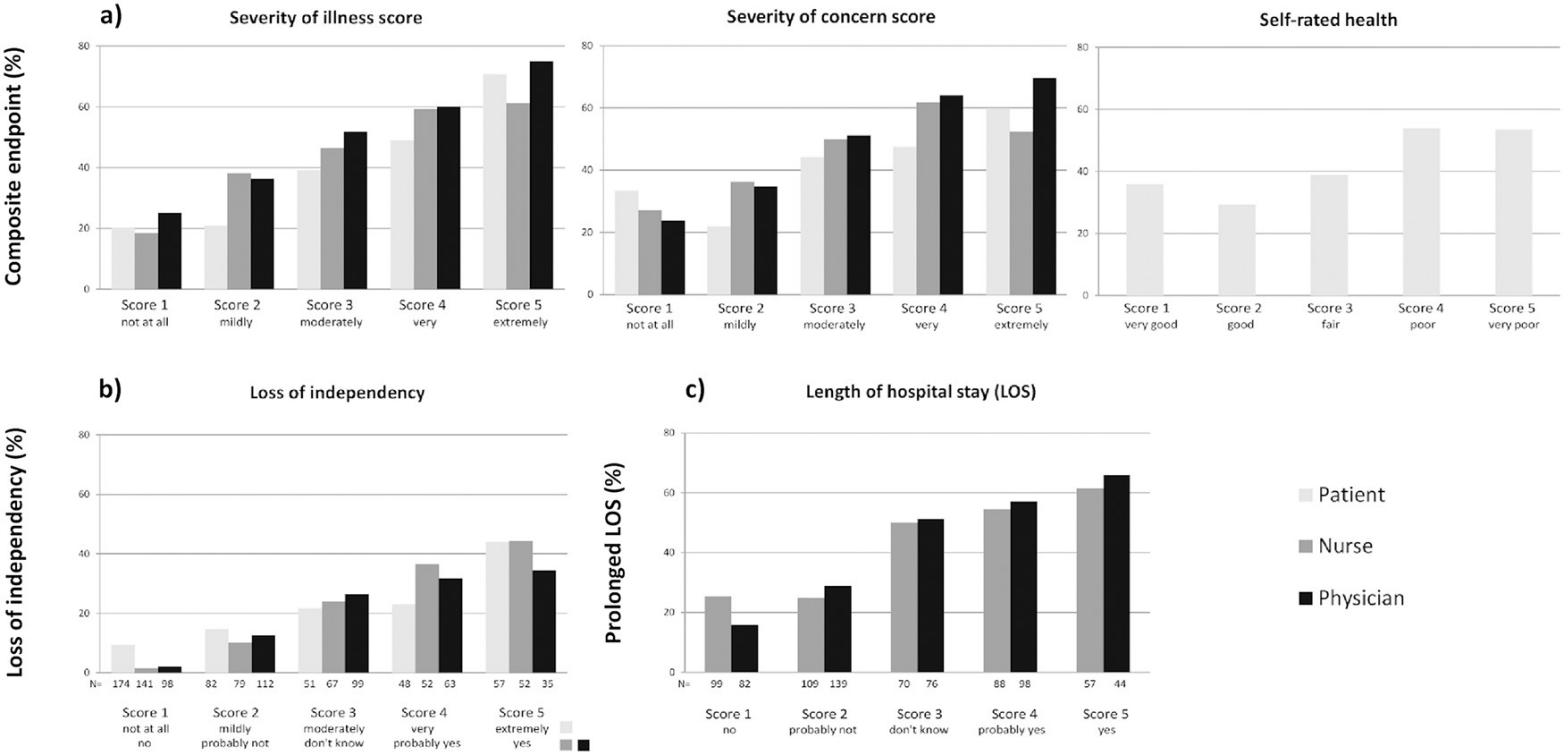
Association between the scores and the composite endpoint (a), the loss of independency score and loss of independent living (b) and the LOS score and prolonged LOS (c).

In total, 66 (11.0%) patients died within 30 days of their ED visit and 263 patients (43.7%) met the composite endpoint (Table 2). For all scores, 30-day mortality increased with increasing value (Fig 2). For the composite endpoint, a similar pattern was seen (Fig 3a).

**Table 2:** Outcomes of study population^a^.

Outcomes	All patients N = 602
30-day all-cause mortality	66 (11.0)
ICU or MCU admission	26 (4.3)
Prolonged LOS	184 (40.4)^b^
Loss of independent living	76 (18.0)^b^
Readmission within 30 days of discharge	83 (15.7)^b^
Composite endpoint	263 (43.7)

Abbreviations: ICU, intensive care unit; LOS, length of hospital stay; MCU, medium care unit

^a^ Values are numbers (percentages) unless stated otherwise

^b^Denominator for prolonged LOS: 456; for loss of independent living: 422 and for readmission: 527

For 30-day mortality, the AUCs were lower for the patients’ disease perception and self-rated health scores (AUCs ranging from 0.64 to 0.69) than for the clinical impression scores of nurses and physicians (AUCs ranging from 0.71 to 0.75, Table 3). The nurses’ and physicians’ severity of concern scores yielded the highest AUCs (0.75; 95% CI 0.68–0.81 for both) and both were significantly higher than the patients’ severity of concern score. For all scores, AUCs for mortality were higher than those for the composite endpoint (AUCs ranging from 0.60 to 0.67).

**Table 3:** Predictive ability of the scores.

	Without cut-off value	With cut-off value						
Corresponding score and outcome	AUC (95% CI)	Cut-off	Sensitivity (%)	Specificity (%)	PPV (%)	NPV (%)	LR+	LR-
**Mortality**								
Severity of illness score								
Patient	0.69 (0.62–0.76)	4	78.1	48.9	15.6	94.9	1.5	0.5
Nurse	0.71 (0.64–0.78)	4	48.4	82.1	25.6	92.6	2.7	0.6
Physician	0.72 (0.65–0.79)	4	47.0	85.9	29.2	92.9	3.3	0.6
Severity of concern score								
Patient	0.64 (0.56–0.71)	4	73.4	52.8	15.8	94.3	1.6	0.5
Nurse	0.75 (0.68–0.81)^a^	3	85.9	54.7	19.4	96.8	1.9	0.3
Physician	0.75 (0.68–0.81)^a^	4	54.5	83.1	28.6	93.6	3.2	0.6
30-day SQ								
Nurse	0.73 (0.66–0.80)	4	56.9	82.1	26.1	94.5	3.2	0.5
Physician	0.74 (0.68–0.81)	4	49.1	87.6	31.0	93.8	4.0	0.6
Self-rated health score								
Patient	0.67 (0.58–0.75)	4	63.3	65.2	16.3	94.3	1.8	0.6
**Composite Endpoint**								
Severity of illness score								
Patient	0.67 (0.62–0.71)	4	67.6	56.6	55.1	68.9	1.6	0.6
Nurse	0.63 (0.58–0.72)	3	71.0	47.2	51.0	67.7	1.3	0.6
Physician	0.65 (0.61–0.69)	3	64.8	59.5	55.4	68.5	1.6	0.6
Severity of concern score								
Patient	0.63 (0.58–0.67)	4	60.3	58.1	53.2	65.0	1.4	0.7
Nurse	0.63 (0.58–0.67)	3	62.0	59.6	54.4	66.9	1.5	0.6
Physician	0.67 (0.62–0.71)	3	65.1	61.9	57.0	69.6	1.7	0.6
Self-rated health score								
Patient	0.60 (0.55–0.65)	4	48.3	70.2	53.7	65.5	1.6	0.7
**Loss of independent living**								
Loss of independency score								
Patient	0.69 (0.62–0.76)	3	62.7	76.7	30.1	89.1	2.7	0.5
Nurse	0.81 (0.75–0.86)^a^	4	80.8	77.2	40.4	95.5	3.5	0.3
Physician	0.72 (0.66–0.78)	4	66.7	74.6	28.8	92.4	2.6	0.5
**Prolonged LOS**								
LOS score								
Nurse	0.67 (0.61–0.72)	4	61.5	71.6	57.2	75.0	2.2	0.5
Physician	0.70 (0.65–0.75)	4	61.6	74.7	59.9	76.0	2.4	0.5

Abbreviations: AUC, area under the curve; CI, confidence interval; PPV, Positive Predictive Value; NPV, Negative Predictive Value; LR+, positive likelihood ratio; LR-, negative likelihood ratio; SQ, surprise question; LOS, length of hospital stay

^a^AUC is significantly higher than the other AUCs for the same score

For patients, the scores yielded higher sensitivity (around 75%) than specificity (around 50%) with respect to 30-day mortality (Table 3). For nurses and physicians, sensitivity was lower (about 50%) than specificity (around 85%). The only exception was the nurses’ severity of concern score, revealing high sensitivity (85.9%) and low specificity (54.7%). NPVs were high (around 94%) for all scores whereas PPVs were higher for nurses and physicians than for patients (around 25–30% and 15% resp.). Positive likelihood ratios(LRs+) for 30-day mortality were higher for the scores of nurses and physicians than for patients, while negative likelihood ratios(LRs-) were comparable between the three groups.

For the composite endpoint, the patients’ scores yielded a lower sensitivity but higher specificity compared to the prediction of mortality. For both nurses and physicians, sensitivity was higher for the composite endpoint than for mortality, while specificity was lower (around 60%). For the composite endpoint, LRs+ were lower for nurses’ and physicians’ scores than for mortality.

### Predictive ability of the scores for loss of independent living and prolonged LOS

Loss of independent living was documented in 76 (18.0%) patients and 184 (40.4%) patients were admitted longer than 7 days (prolonged LOS, Table 2). With increasing scores, more patients met these endpoints (Fig 3b and 3c). The AUC of the nurses’ loss of independency score was very good (AUC: 0.81), and significantly higher than the patients’ and physicians’ score (AUC: 0.69 and 0.72 resp., p-value <0.05). For the other scores, AUCs were lower (around 0.70).

Both sensitivity (80.8%) and specificity (77.2%) were high for the nurses’ loss of independency score and this resulted in a LR+ of 3.5 and LR- of 0.3. For the other scores, both sensitivity and specificity were lower.

### Interrater reliability

The interrater reliability between patients and the healthcare professionals was poor with intraclass correlation coefficients (ICCs) ranging from 0.12 to 0.37 for the three scores (Table 4). We obtained a fair interrater reliability between nurses and physicians (ICCs around 0.50) for all of the four scores, except for the SQ that yielded a poor ICC of 0.33.

**Table 4:** Intraclass correlation coefficients with 95% CI for the different scores.

Comparison	ICC (95% CI)
**Patient-Nurse**	
Severity of illness score	0.31 (0.13–0.45)
Severity of concern score	0.20 (0.07–0.32)
Loss of independency score	0.28 (0.20–0.36)
**Patient-Physician**	
Severity of illness score	0.30 (0.05–0.49)
Severity of concern score	0.23 (0.09–0.34)
Loss of independency score	0.37 (0.30–0.44)
**Nurse-Physician**	
Severity of illness score	0.50 (0.43–0.56)
Severity of concern score	0.46 (0.40–0.53)
30-day SQ	0.33 (0.26–0.40)
Loss of independency score	0.50 (0.43–0.56)

Abbreviations: ICC, intraclass correlation coefficient; CI, confidence interval; SQ, surprise question

### Predictive ability of combined scores

Combination of scores of patients, nurses and physicians yielded higher LRs+ and LRs- when there was agreement (Table 5). When extreme scores (i.e. score 1 or score 5) of patients, nurses and physicians were combined, LRs highly improved. The extreme scores, however, were only encountered in a limited number of patients, except when score 1, “Yes, I would be surprised”, was given for the SQ (n = 188).

**Table 5:** Likelihood ratios of combined scores for 30-day mortality.

Combination of scores				n	LR	Observed Mortality (%)
**Severity of illness score**						
Agreement						
	Patient +	Nurse +	Physician +	47	6.0	42.6
	Patient -	Nurse -	Physician -	214	0.3	3.7
		Nurse +	Physician +	58	5.1	39.7
		Nurse -	Physician -	399	0.5	6.3
		Nurse score 5	Physician score 5	7	47.0	85.7
		Nurse score 1	Physician score 1	33	0.3	3.0
Disagreement						
		Nurse +	Physician -	63	1.1	12.7
		Nurse -	Physician +	45	1.7	17.8
**Severity of concern score**						
Agreement						
	Patient +	Nurse +	Physician +	68	5.9	42.6
	Patient -	Nurse -	Physician -	156	0.2	1.9
		Nurse +	Physician +	94	4.4	36.2
		Nurse -	Physician -	256	0.3	3.1
		Nurse score 5	Physician score 5	8	23.5	75.0
		Nurse score 1	Physician score 1	43	0.4	4.7
Disagreement						
		Nurse +	Physician -	188	1.0	11.2
		Nurse -	Physician +	26	0.3	3.8
**Surprise question**						
Agreement						
		Nurse +	Physician +	34	10.7	52.9
		Nurse -	Physician -	319	0.4	
		Nurse score 5	Physician score 5	3	∞	100
		Nurse score 1	Physician score 1	188	0.1	1.6
Disagreement						
		Nurse +	Physician -	57	1.6	14.0
		Nurse -	Physician +	36	0.9	8.3

Abbreviations: n, number of patients with the corresponding scores; LR, likelihood ratio

Disagreement between nurses and physicians was less common (around 20%) than agreement. When nurses and physicians disagreed, LRs were low and around 1.0–1.5, except for the severity of concern score, where the LR was 0.3 when a physician was concerned but the nurse disagreed.

### Selection bias

Table 6 shows the patient characteristics and outcomes of the included and non-included patients during study recruitment. The two groups were comparable regarding age and sex but clinical signs of cognitive impairment and delirium were less frequently present in included patients (29.1 vs. 38.6%, resp.). Outcomes of included and non-included patients did not differ, although 30-day mortality was slightly lower for the included patients.

**Table 6:** Overview of characteristics and outcomes of included and non-included patients^a^.

	Included Prospectively analysed N = 602	Non-included Retrospectively analysed N = 200	Total All possible candidates N = 2109
Demographics			
Age (years), median (IQR)	79 (73–85)	78 (72–85)	78 (71–84)
Male	311 (51.7)	100 (50.0)	1023 (48.5)
Living community dwelling	522 (86.7)	155 (78.7)	
Living in nursing/care home	50 (8.3)	23 (11.6)	
Comorbidity and cognitive functioning			
Charlson comorbidity index score	2 (1–3)	2 (1–3)	
Cognitive impairment or delirium	167 (29.1)	66 (38.6)	
Outcomes			
30-day mortality	66 (10.9)	30 (15.0)	
Composite endpoint	263 (43.7)	84 (42.0)	

^a^Values are numbers (percentages) unless stated otherwise. Incomplete data in the prospective and retrospective cohort for: cognitive impairment or delirium (n = 28 and n = 29, resp.) and outcomes (n = 0 and n = 1, resp.).

## Discussion

To the best of our knowledge, this is the first prospective study examining the discriminatory value of clinical intuition—disease perception, self-rated health and the first clinical impression—with respect to short-term mortality and other adverse outcomes in older ED patients. We found that 30-day mortality can be predicted by application of the clinical intuition questionnaire for patients, nurses and physicians early after arrival in the ED. The best discriminatory values were encountered for the nurses’ and physicians’ severity of concern score (AUCs of 0.75). When patients and professionals were in agreement, diagnostic accuracy further improved. The discriminatory value of the first clinical impression, disease perception and self-rated health for the composite endpoint was lower, but still sufficient (AUCs ranging from 0.60 to 0.67), compared to the prediction of 30-day mortality. Loss of independent living was best predicted by nurses.

### Comparison with previous studies

Our findings concerning the discriminatory value of the first clinical impression of nurses and physicians are in line with other studies. A Swiss study [7] showed that the first clinical impression of physicians was predictive for 30-day mortality with an AUC of 0.66, which was substantially lower compared to our results (AUCs ranging from 0.71 to 0.75). This difference may be explained by the use of a different scoring system, inclusion of patients with non-specific complaints or selective exclusion of seriously ill patients in the Swiss cohort. In a Danish study [6], nurses and physicians in a medical admission unit both predicted in-hospital mortality adequately (AUC 0.82 for nurses and 0.76 for physicians). In line with our study, they found a higher accuracy when both nurses and physicians agreed (76%). This finding supports the importance of teamwork in emergency care. An explanation for the high AUCs found in this Danish study may be that in-hospital mortality was predicted when results of the first evaluation and diagnostics were available. We, however, decided to investigate the *first* clinical impression of nurses and physicians immediately after arrival in the ED at the moment that major decisions about prioritization of diagnostics and treatments have to be made. This judgement turned out to be accurate, even in a relatively unexperienced group of professionals.

The SQ turned out to be to be a good predictor of 30-day mortality. In a recent meta-analysis [15], the discriminatory value and specificity of the SQ for 30-day mortality in one study with septic ED patients was lower than in our study (AUC of 0.59 and specificity of 69%). This may be explained by the fact that in-hospital and not 30-day mortality was used as outcome. Furthermore, it might be possible that differences between sepsis patients who are at risk of dying and those who are not are hard to make. Based on our data, we conclude that the 30-day SQ predicts 30-day mortality accurately in older medical ED patients.

The discriminatory value of the three scores regarding 30-day mortality was lower for patients than for professionals but this difference was, except for the severity of concern score, not significant. NPVs were high (around 94%) for patients and professionals and LRs- were low when patients and professionals agreed. This indicates that when scores are negative, especially when in agreement, there is a low probability of mortality. This finding can support adequate decision making about dismission of patients from the ED. We recorded higher specificity, PPVs and LRs+ for nurses and physicians than for patients. This is not surprising, as professionals are confronted with acutely ill patients on a daily basis. The observation that patients frequently scored themselves very or extremely ill and that they were severely concerned is in line with this assumption. We found a poor interrater reliability between patients and professionals. These findings match with another Swiss study [10] that found lower discriminatory values for the assessment by patients than by nurses or physicians. This Swiss study also found a poor interrater reliability between patients and professionals (ICC of 0.17 for patient/nurse and 0.07 for patient/physician). Nevertheless, when patients agreed with professionals, discriminatory values for mortality improved. In addition, sensitivity was higher for the patients’ scores compared to that of professionals, which may support the importance of involvement of patients in clinical decision making.

In this study, we found lower discriminatory values for the composite endpoints than for mortality. This may be explained by the fact that the scores are based on the intuitive feeling on whether a patient is at risk of dying instead on being at risk of other outcomes. However, loss of independent living was accurately predicted by nurses (AUC 0.81) indicating that this specific score can be used as a screening instrument by nurses, in order to deliver appropriate care.

### Study limitations

Our study has several limitations. First, we used scores, which have not been previously validated, including the severity of illness and severity of concern score. However, a comparable 9-point [5] and 10-point score [25] was used in other studies, showing a similar association and discriminatory value for mortality. Nevertheless, future studies may be helpful to externally validate our scores and to test reproducibility. Second, both the nurses and physicians participating in this study had only a few years of professional experience, a factor that may be different in other EDs, hospitals or countries. It is possible that intuition is more predictive in more experienced professionals. Third, we tested the discriminatory value of the *first* clinical impression of nurses and physicians. We do not know whether this discriminatory ability improves after physical examination and diagnostics or in a later stage during hospital admission. On the other hand, we are convinced that a quick judgement in the ED is essential to optimize safe and personalized care. In the fourth place, our results may have been biased because of selective inclusion of patients since many patients were not asked to participate. For this reason we retrospectively compared the data of non-included candidates with our study population and did not find evidence for selection bias.

### Conclusions

In conclusion, clinical intuition—disease perception, self-rated health and first clinical impression—predicts mortality and other adverse outcomes in older ED patients. Intuition is inexpensive and can identify severely ill patients in an early stage, which may contribute to timely and personalised treatment and aftercare. Diagnostic accuracy improves when patients and professionals are in agreement, which may support the importance of shared decision making. We think that professionals should be aware that their first impression is a valuable tool to predict clinical sequelae and can help them to apply personalized medical care. Studies are needed to validate our scores, to test their reproducibility and to test their ability to improve clinical outcome or well-being in older ED patients.

## Supporting information

S1 File Questionnaires of patients, nurses and physicians. (DOCX)

S2 File Dataset clinical intuition. (XLSX)
