# Supplementary material for: Short-term mortality in older medical emergency patients can be predicted using clinical intuition: A prospective study
Source: PLoS One. 2019 Jan 2;14(1):e0208741. doi: 10.1371/journal.pone.0208741 (PMC6314634; doi:10.1371/journal.pone.0208741)
Supplement: S1 File — (DOCX) [file pone.0208741.s001.docx]

**S1 File: Questionnaires of patients, nurses and physicians**

**30-day mortality in older medical emergency patients can be predicted using clinical intuition: a prospective study**

Noortje Zelis, MD^1,4^, Arisja N Mauritz, MD^2^, Lonne I J Kuijpers, MD^1^, Jacqueline Buijs, MD PhD^1^, Peter W de Leeuw, MD PhD^1,2,4^ and Patricia M Stassen, MD PhD^2,3^

**Table of content**

Questionnaire 1: Emergency department questionnaire for the patient or caregiver…………p2

Questionnaire 2: Emergency department questionnaire for the nurse……………………………….p4

Questionnaire 3: Emergency department questionnaire for the physician……………….…………p6

**Questionnaire 1: Emergency department questionnaire for the patient or caregiver**

**
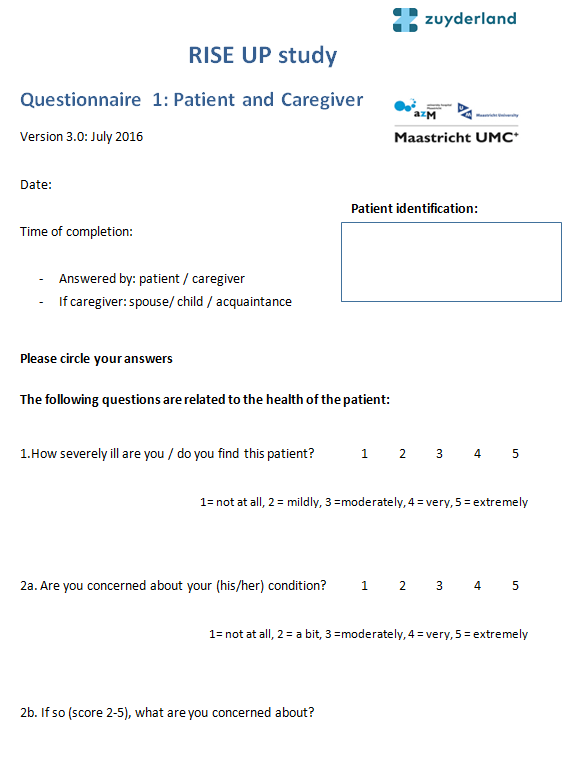
**

**
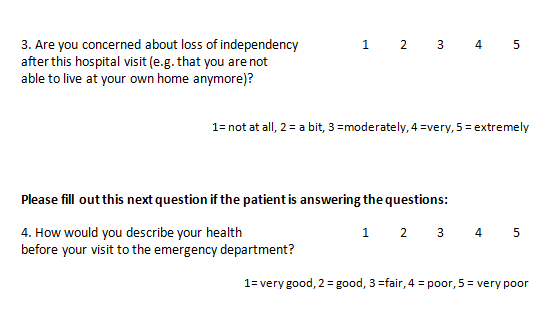
**

**Questionnaire 2: Emergency department questionnaire for the nurse**

**
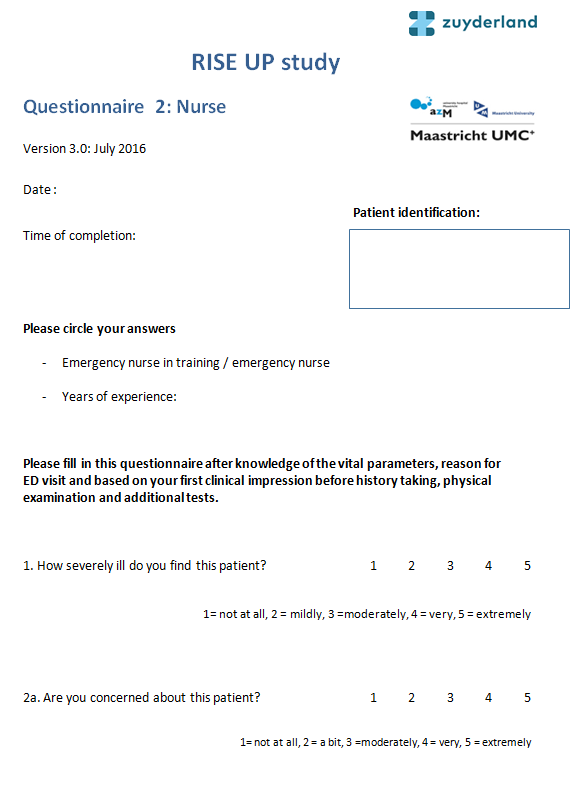
**

**
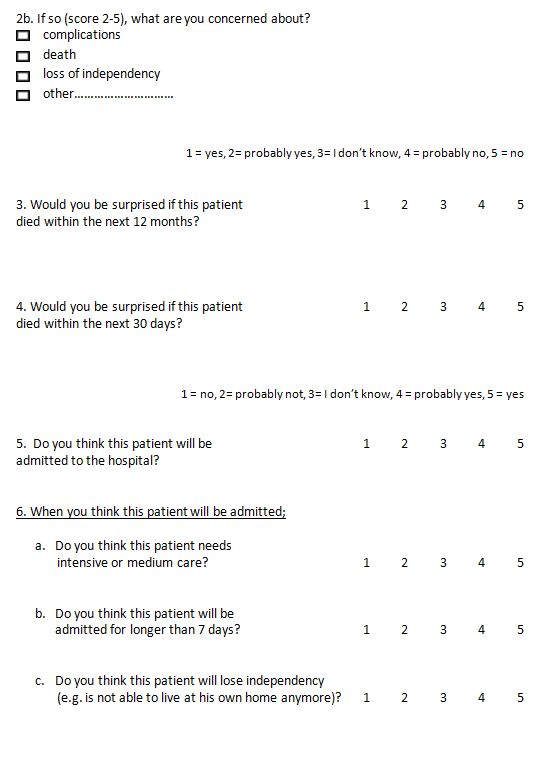
**

**Questionnaire 3: Emergency department questionnaire for the physician**

**
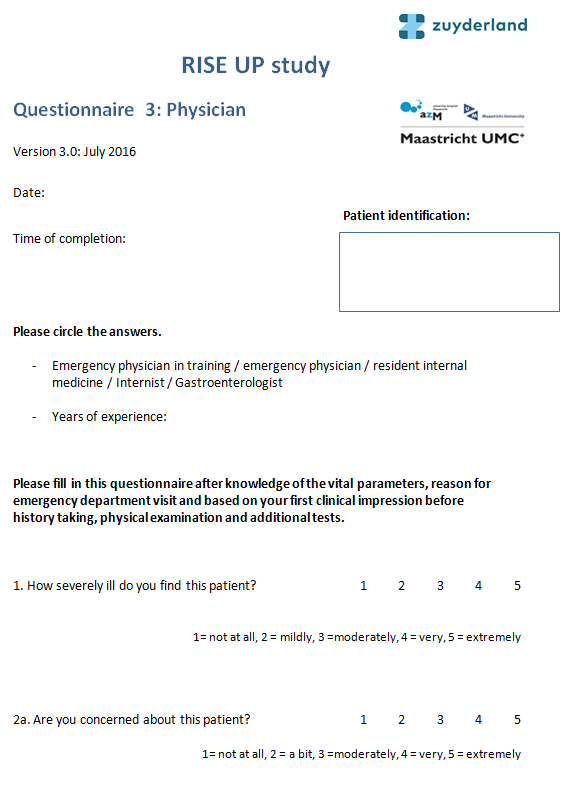
**

**
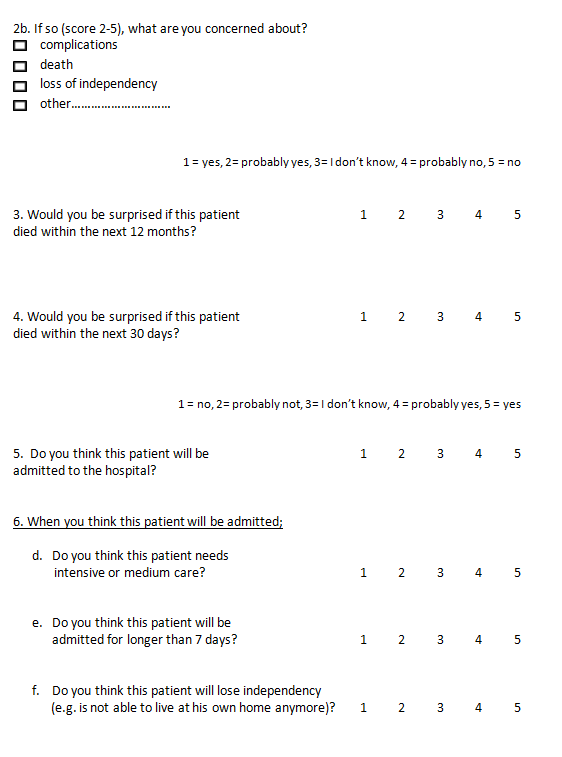
**
